# Detection of fractured zones, faults, and cavities by high resolution muon tomography in the Buda Hills

**DOI:** 10.1038/s41598-025-02510-0

**Published:** 2025-05-20

**Authors:** László Balázs, Gergő Hamar, Ádám Csicsek, Gergely Surányi, Dezső Varga

**Affiliations:** 1 HUN-REN Wigner Research Centre for Physics, Hungarian Research Network, 29-33 Konkoly-Thege Miklós Str., Budapest, 1121 Hungary; 2 Department of General and Applied Geology, Eötvös Loránd University, Pázmány Péter sétány 1/A, Budapest, 1117 Hungary

**Keywords:** Muon tomography, Muography, Fracture detection, 3D inversion, Bayesian, Particle physics, Techniques and instrumentation, Geology, Geophysics

## Abstract

The high-penetration cosmic-ray muons can be used for scanning and exploring the structure of large geological formations, up to several hundred meters of thickness. Since the attenuation of the muon flux as it passes through the object depends on the density of the rock mass being scanned, measurements with high resolution muon detectors (muography) in suitable arrangement allow reconstruction of three-dimensional density distribution (muon tomography). The estimated density distributions can be used to infer the geological structure of the screened rock mass if the density inhomogeneity reflects it. The muographic method is extremely efficient because the mapping is performed along approximately straight lines, similar to X-ray or CT scans, since the deflection of high-energy muon trajectory due to interaction with the rock material is almost negligible. This ensures that density inhomogeneities can be determined with much sharper contours and higher accuracy than with other geophysical methods. In this paper we present a muography based high precision identification of a fractured zones and cavities which explorations are challenging to surface geophysical methods. The applicability of muon tomography is demonstrated in an area which is built up by Mesozoic carbonates (Buda Hills, Hármashatárhegy Range in Hungary) with variable topography, where the dominant directions and trends of the fault systems and fracture network are well known, which allows the tomographic results to be controlled. This case study demonstrates that muographic method is capable to obtain an accurate 3D picture of the density anomalous fracture system in carbonates for a rock thickness up to 100 m, that can very useful and unique contribution to geological interpretation and geological model building.

## Introduction

In the atmosphere, high-energy cosmic rays produce secondary particle showers. The essential components of this shower are muons^1,2^, which provide a natural source of near-constant^3^ intensity radiation for ”scanning” rock masses. Muography^4^ based on the detection of high-energy relativistic muons penetrating the rock masses under investigation up to several hundred meters thick is in some cases a unique geophysical tool for the exploration of geological structure using tomographic reconstruction of density distributions^5–7^. Due to their relatively large mass and high energy, muons suffer little deflection^8–10^ during interactions with rock matter, and therefore their trajectories are essentially straight. The rock density distribution along the muon trajectory, or even better the orbital integral of the density (density-length or opacity), is responsible for the attenuation^11^ of the penetrating muon flux. This phenomenon gives the basis for muographic methods and tomography. (The cosmogenic muon flux attenuation mechanism is discussed in detail by Lesparre et al.^6^.) The angular and energy distribution of the cosmic muon flux on the surface (as a reference distribution) is also crucial for the application of muography^12–14^. This information can be used to estimate the muon flux attenuation through a given rock mass. (Muon flux attenuation can be written as a monotonic function of density-length, which is discussed by several authors: Lesparre^6^, Bugaev et al.^13^ and Reyna^15^.) The way of muographic mapping, the almost straight path of high-energy muons, ensures the unique efficiency in detecting cavities, fracture systems, and mineral deposits (e.g., ore bodies) of different densities. For other geophysical methods, the mapping may depend strongly on the medium, the spatial distribution of the rock physics properties under investigation (e.g., the dependence of the elastic wave path on the seismic velocity distribution or the dependence of the current density field on the resistivity distribution for electrical tomography methods).

**Fig. 1:**
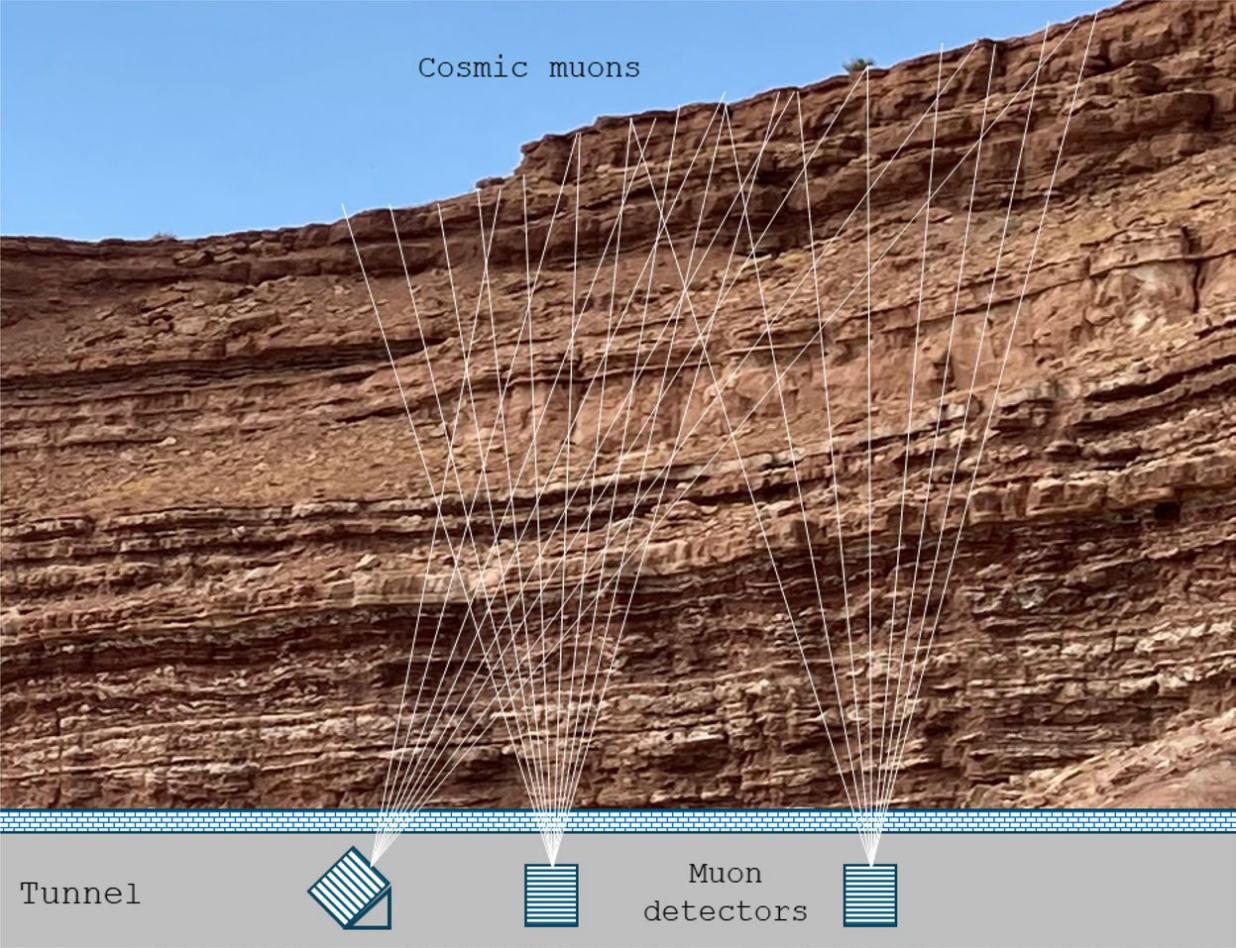
Schematic drawing of subsurface muographic measurements. The mapping cones show the angular range from which the detected muon intensity is being processed separately for tomographic evaluation (angular bin). The overlapping 3D mapping cones are essential for the proper tomographic reconstruction of the 3D density distribution.

The muographic method also requires the development of specific measurement tools. The special multilayer detector^16,17^ applied to track individual muon particles determines the parameters of the muon trajectories (azimuth, zenith). This detector design ensures direction-sensitive detection and also the selectivity for muons. By extrapolating the intra-detector muon trajectories to the rock body under investigation, it is possible to determine for which rock range a given measurement contains information (Fig. 1). The spatial distribution of the rock density can be estimated from a measured muographic data set (position- and direction-dependent muon intensities) derived from overlapping measurements with several direction-sensitive detectors (muon tomography).

**Fig. 2:**
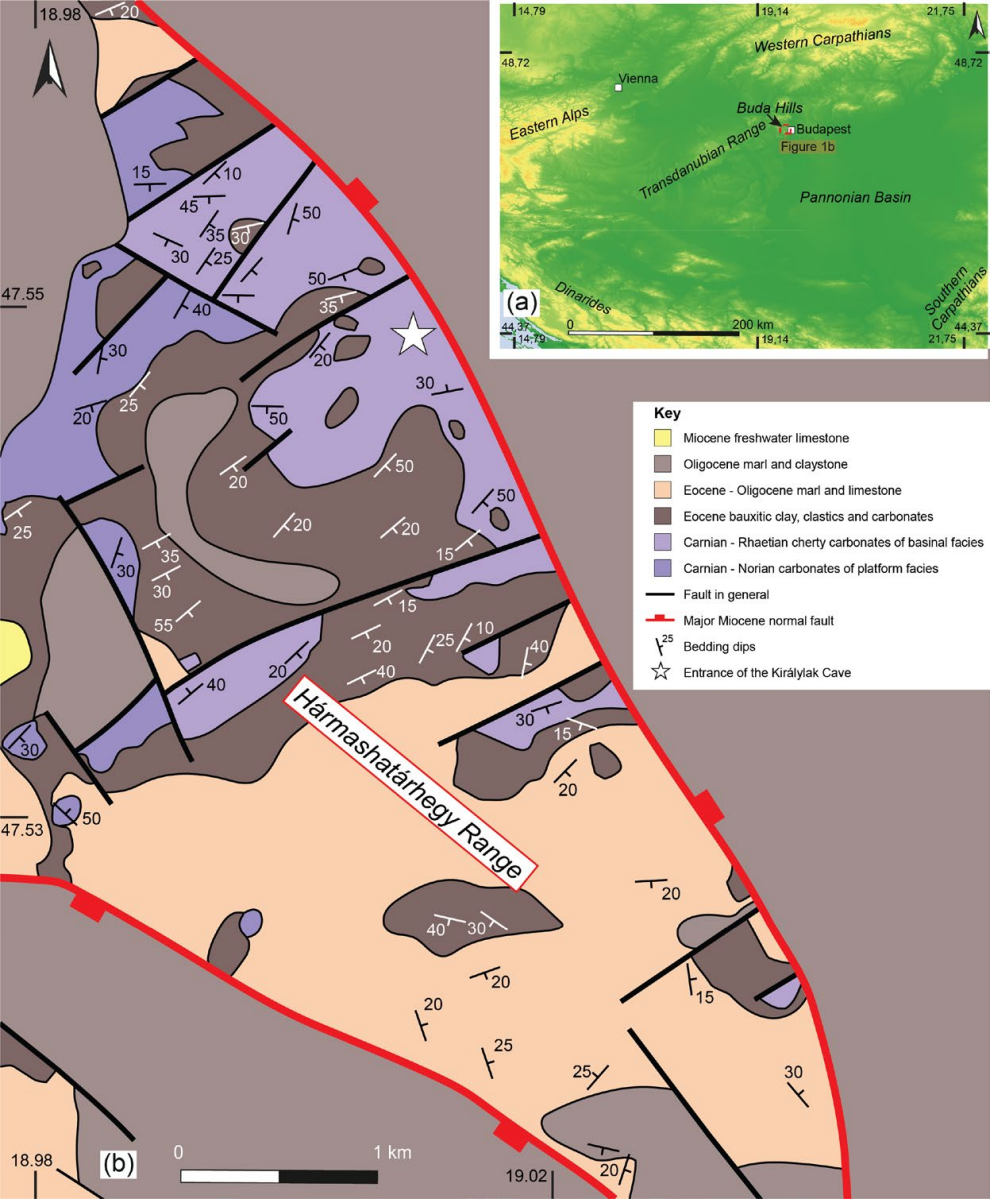
(**a**) Map showing the location of the Buda Hills in the Pannonian Basin (**b**) Pre-Quaternary geological map of the Hármashatárhegy Range and its surroundings. (Modified after Gyalog^18^, Wein^19^).

Our case study demonstrates the usefulness of tomography in a fractured carbonate (Triassic cherty dolomite) area (Fig. 2b) where the dominant direction of the fault and fracture system is relatively well known and can be spatially traced in some places using local outcrops and caves. The information provided by muography can be useful in geological, hydrogeological modeling, cave exploration, or in identifying geotechnical problems such as the risk of landslides.

The geological applications of muography^20^ go back about a decade. The special requirements in geological research have necessitated the development of special direction-sensitive muon detectors (muon telescope). High-quality 3D tomographic reconstruction requires large numbers and high-resolution measurement data sets, and hence long measurement times, so the number of published applications in this area is relatively small. For vulcanological purposes, French^21^ and Japanese^22,23^ authors have reported muographic measurements suitable for high-resolution 3D tomography. In these cases, surface muographic measurements were used to obtain detailed information on the inner structure of volcanoes. Cavity research with 3D reconstruction using subsurface measurements has been carried out by Cimino et al.^24^ and Borselli et al.^25^ at shallow depths, mainly for archaeological purposes. The subsurface muographic method has also been successfully used in mining-related exploration to delineate ore bodies with high density contrasts to bedrock; a real example has been shown e.g. by Schouten and Lendru^26^, Zhang et al.^27^ or Beni et al.^28^. The researchers of Wigner RCP (Hungary) had previously carried out the identification of the fracture zone using proprietary high-resolution detectors using a series of 2D tomographic reconstructions of subsurface measurements^29^ to approximate the geological structure in 3D in greater depth. The muon telescope used was developed specifically for geological research^30^. Our current study presents recent advances in subsurface measurements and their processing, and new results which have been achieved by full 3D inversion of a large dataset of muographic measurements to determine the geometry of the subsurface fracture and cavity system. The development of detector systems and related software is now ongoing. Muography as a geophysical method is at the beginning of its history, so further significant improvements in technology and data processing are expected, although the information it provides is already uniquely useful.

### Geological setting

The Buda Hills are located in the central part of the Pannonian Basin and represent the northeastern segment of the Transdanubian Range (Fig. 2a). The complex geological evolution of the study area was characterized by the opening and closure of two oceanic domains, the eastern Triassic-Cretaceous Neotethys and the western Jurassic-Tertiary Alpine Tethys Oceans, and the subsequent formation of the Neogene Pannonian Basin^31–35^

Regional considerations suggest that the basement of the Transdanubian Range is composed of Variscan metamorphites and their Paleozoic cover^36,37^. During the Triassic-Early Jurassic, the area was part of the proximal Adriatic margin of the Neotethys^31,33^ which was affected by several stages of rifting during Late Permian and Triassic times. The oldest part of the Neotethyan succession, which is exposed in the southern and northern part of the Buda Hills, is composed of Upper Anisian-Ladinian thick-bedded dolomite^38^. From the Carnian onwards, an extensive carbonate platform developed on the margin and a thick succession of platform carbonates such as dolomite and limestone, deposited in the area of the Buda Hills^39^.

**Fig. 3:**
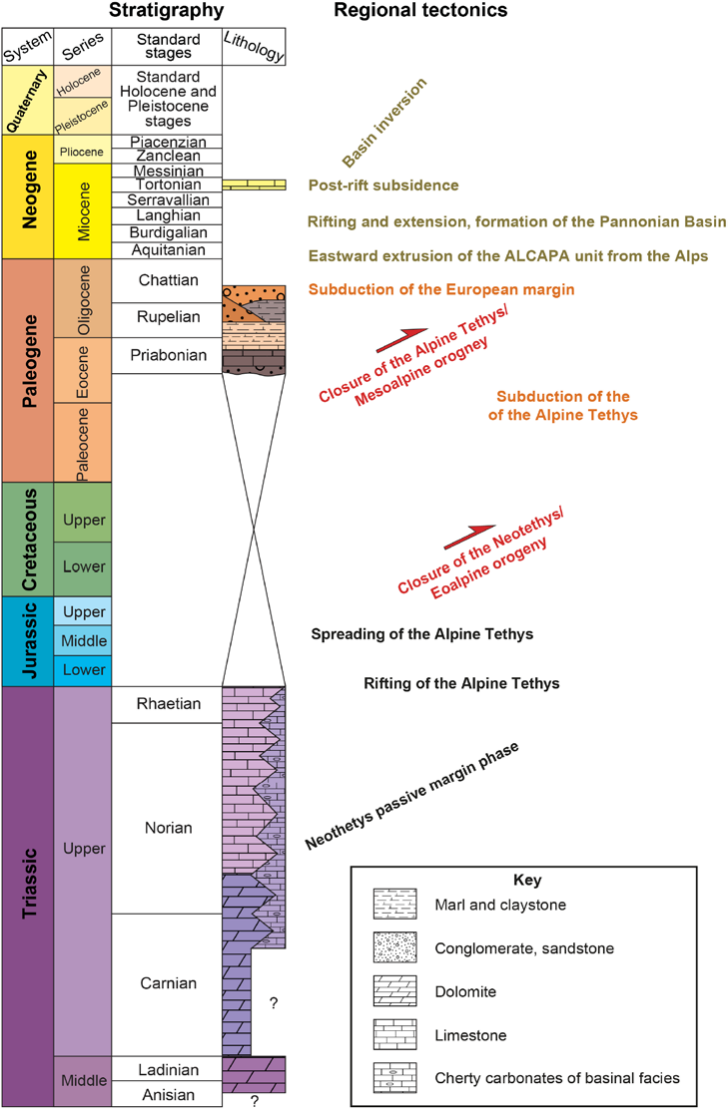
Lithostratigraphic column of the study area and regional tectonic events in the Transdanubian Range. (Modified after Karádi^44^, Babinszky^47^ and Wein^19^).

However, the development of the extensive carbonate platform was interrupted by a second phase of rifting during the Late Triassic, and roughly NW-SE trending extensional faults defined a distinctive paleogeographic configuration^40–42^. Sedimentation was characterized by the deposition of basinal and to-of-slope facies of cherty carbonates in intra-platform basins whereas in the central part of the Buda Hills the development of the carbonate platform was continuous^19,42^. In the study area, the Hármashatárhegy Range, the Upper Triassic succession is made up of Carnian-Norian platform carbonates and Carnian/Rhaetian cherty carbonates of basinal facies^43,44^ (Figs. 2b and 3). Continuous extension affected the area during the Early Jurassic^40,45,46^.

Rifting resulted in the separation of the Adriatic plate from Europe during the Early Jurassic and the opening of the Alpine Tethys Ocean during the Middle Jurassic^32–35,48^. The Jurassic of the Transdanubian Range is mainly composed of a thin and reduced succession of pelagic carbonates^49^. The Jurassic section gradually passes up into Lower Cretaceous clastics which was deposited in a flexural foreland basin. This Early Cretaceous basin developed in the northeast part of the Transdanubian Range due to the closure of the Neotethys Ocean^33,50,51^. However, the above-mentioned Jurassic-Cretaceous succession was eroded in the surroundings of the Buda Hills because of large-scale folding and thrusting related to Austroalpine nappe emplacement (the so-called Eoalpine orogeny) during the middle of the Cretaceous^52–54^. The Transdanubian Range was involved in thick-skinned deformation and became the uppermost nappe of the Austropalpine nappe system^33,55–57^. Nappe emplacement resulted in the formation of the NE-SW trending, large-scale syncline of the Transdanubian Range^51^ whereas E-W trending, north and south verging thrust faults, and associated folds developed in the vicinity of the Buda Hills^19,52,58^. During the Late Eocene the folded Mesozoic succession of the Buda Hills was the subject of marine transgression^59^. Sedimentation occurred in a retroarc flexural basin, the Paleogene Basin, which developed due to the more internal thrust load of the Alpine-Carpathian folded belt (during the so-called Mesoalpine orogeny)^60,61^. At the base of the Eocene sequence, bauxitic clay is exposed which was deposited above the pre-Eocene regional unconformity^62,63^. The flexural basin fill comprises a basal transgressive unit overlain by a mixed marine succession of carbonates and clastics which records the transition from the underfilled flysch to the overfilled molasse basin^60^. According to Fodor et al.^64^ and Fodor, et al.^65^, NE-SW trending folds and reverse faults developed during Late Eocene and Early Miocene times in the Buda Hills and the Hármashatárhegy Range (Fig. 2). The Neogene Pannonian Basin was superimposed over the Alpine nappe system and the Paleogene Basin. The formation of the back-arc basin was accompanied by eastward extrusion from the Eastern Alps and coeval regional extension and sedimentation in a series of half-grabens^66–69^. The Hármashatárhegy Range is bounded to the south and the north by roughly NW-SE trending normal faults (Fig. 2b) which may have been formed during the Neogene regional extension^65^. Miocene sediments deposited mostly along the margins of the Buda Hills and only a thin cover of Late Miocene freshwater limestones was deposited West and southwest of the of the Hármashatárhegy Range^70,71^ (Fig. 2b). Basin inversion started during the Late Miocene in the Pannonian Basin (Fig. 3). Inversion is still continuous today and resulted in the exposure of the Pre-Neogene succession on the basin margins such as the Transdanubian Range^72,73^.

## Results

**Fig. 4:**
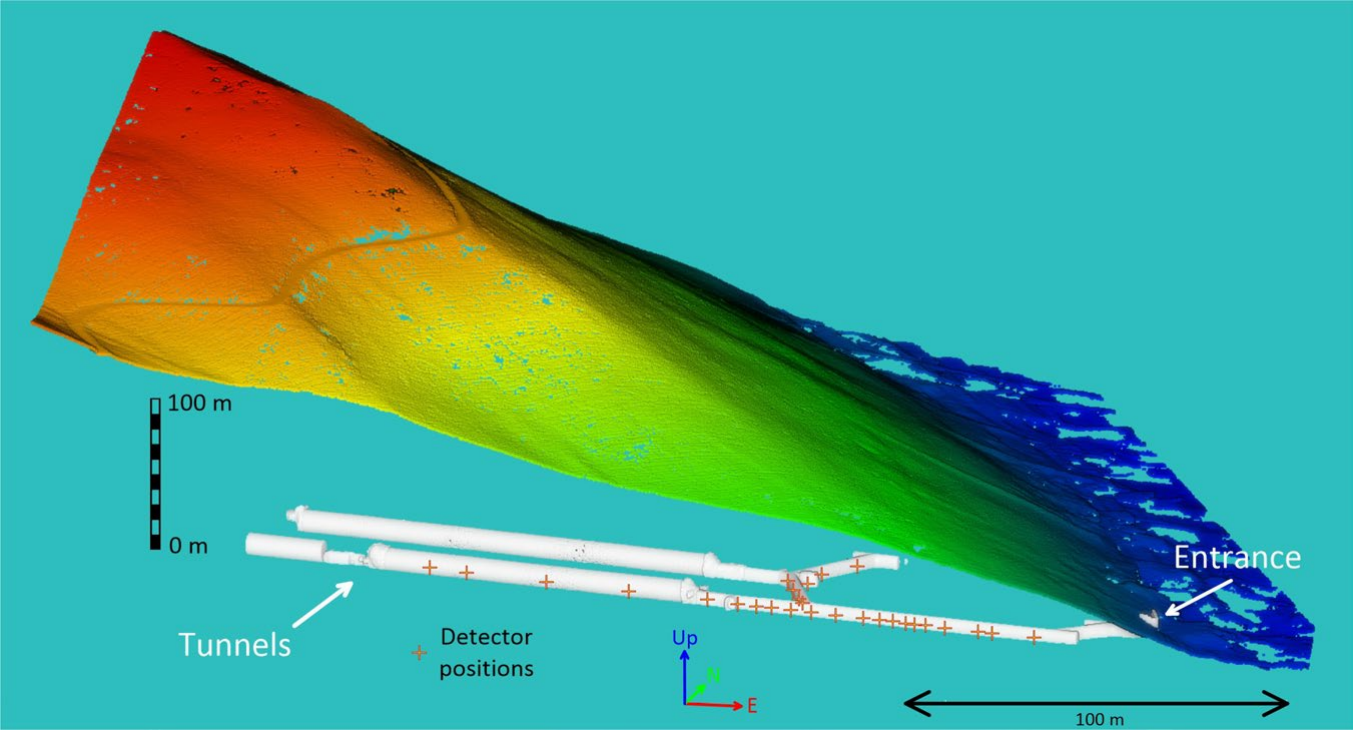
Topography of the research site and the available tunnel therein. The red marks represent the locations of the muograph measurements.

A uniquely large number (during 600 active days, from 43 detector positions recording about 15 million muon tracks have been processed and binned into more than 800 thousand narrow-angle directional muon intensity measurements) of high-angular resolution^30^ subsurface muographic measurements were performed to map the eastern slope of Hármashatár Hill above the Királylaki tunnel (Fig. 4).

**Fig. 5:**
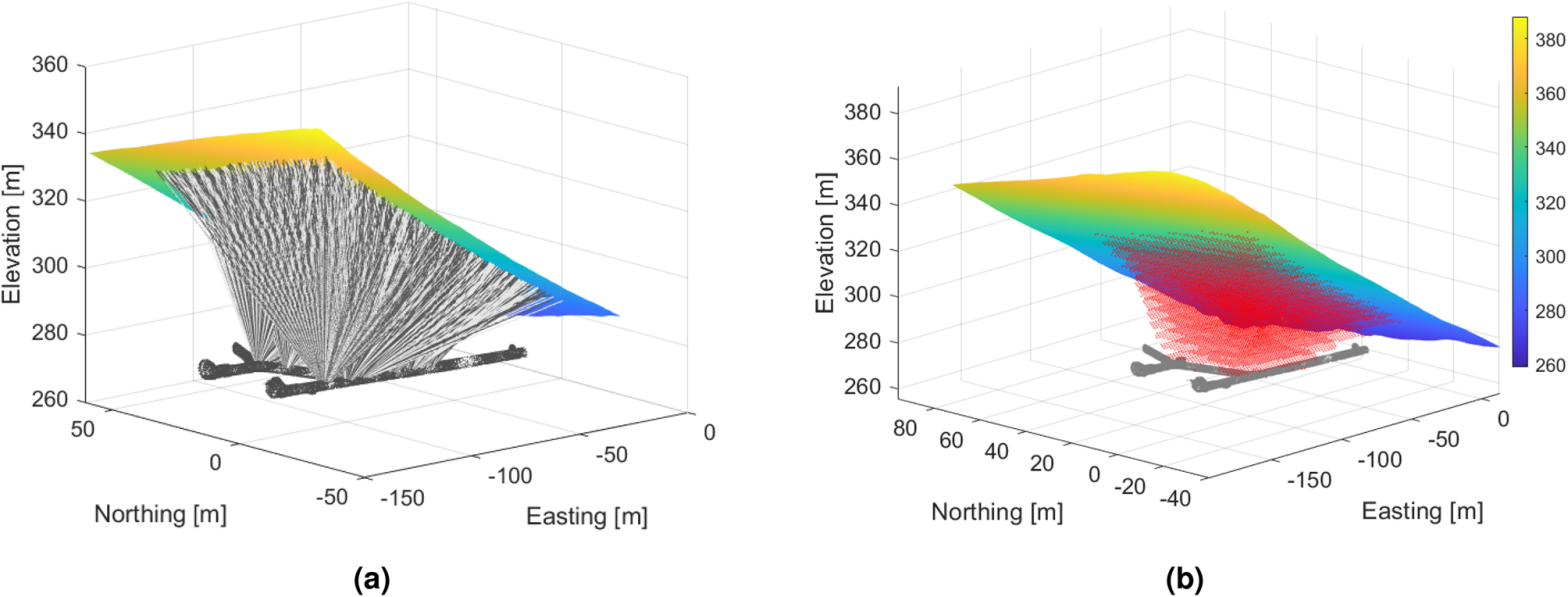
(**a**) The main muon trajectories related to the detectors are shown up to the surface of the hill. These lines outline the sensitive volume of the measurement setup. (**b**) Location of the 3D voxel grid (1.5 m side length) defined for the calculation and visualization of the density distribution. Below the voxel grid, the part of Királylaki-tunnel system is shown, from where the measurements were taken.

High angular resolution^74^ is the key to achieve high spatial resolution tomographic results which can also be used for geological interpretation. The internal structure of the hill studied was only revealed in small excavations and caves. Although DC electrical tomography measurements were made, the high resistivity of the bedrock, surface quality and the topography did not allow any substantive geological conclusions to be drawn. The primary objective of the muographic surveys was to detect the lower density fracture zones and possible cavities in the eastern part of the study area (or possibly the detection of slope debris accumulation), while in the western part a smaller-coverage reconnaissance survey was carried out. The system of measurement positions was designed to meet these aims. In addition to better coverage, the thickness of the dolomite above the detectors is also smaller in the eastern part, resulting in better spatial resolution and accuracy of muography. The quality of the measurements and the high angular resolution allow for a full 3D tomographic reconstruction of the density distribution of the screened rock mass down to a resolution of 1.5 meters.

**Fig. 6:**
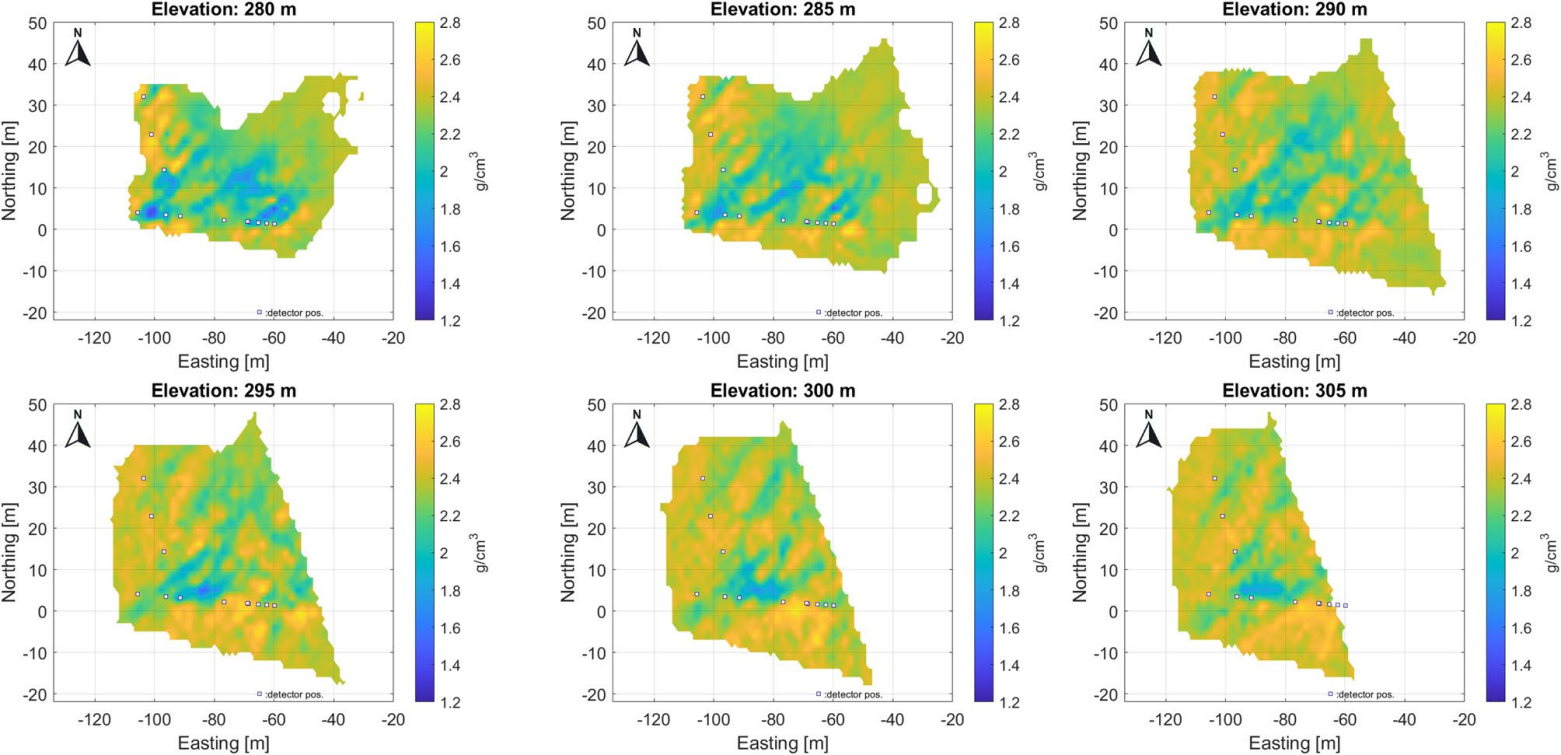
Some horizontal sections from 3D, volumetric tomographic results for the Eastern part of the study area. The low density zones most probably related to the NE-SW fracture zones are well traceable, but possible cavities can also be identified along the fracture system.

The tomography results are represented in discretized form on a 3D grid (voxel network) aligned to the geographic axes, covering the scanned part of the object under investigation. The sensitive volume of tomography is the common part of the voxel network, and the projection cones belong to the measurements (Fig. 5). For higher spatial resolution, the tomographic problem must be solved for 100-200 thousand voxel elements (unknowns in tomographic equations). For illustration, the results of the tomography (as a 3D density distribution) are presented in horizontal sections (Fig. 6).

**Fig. 7:**
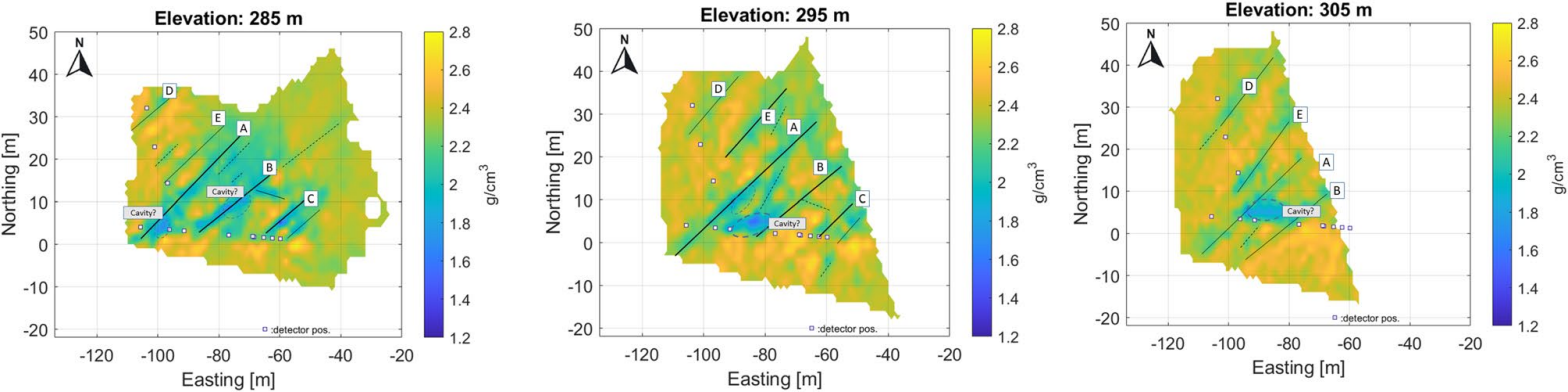
Main, traceable near vertical fracture zones on horizontal section indicated by letters A-G. Above the level 295 m, the small cavity(?) can be identified and traced.

The projected location of detectors in the tunnel at the 270 m level is indicated by small rectangles in each figure. The results are plotted using a local coordinate system aligned with the geographic coordinates from the entrance to the Királylaki tunnel. (The entrance is marked in Fig. 2; latitude: 47.552648 and longitude: 19.010922) Due to the fine resolution, the larger fractures can be identified and are easy to follow from level to level (the main fractures are highlighted and marked with letters A-G in Fig. 7). In summary, the results show that the geometry of the meter-scale fracture system can be well understood.

The successive horizontal sections clearly show a series of near-vertical fracture zones with a typical NE-SW direction, which is the dominant tectonic direction in the area (Fig. 7).

Based on their strike, length, and the age of the deformed succession, we suggest that the identified NE-SW trending fault zones of the Hármashatárhegy Range may have been formed during Late Eocene - Early Miocene times and correspond to the NE-SW trending faults described by Fodor et al.^64^ and Fodor et al.^65^.

When comparing the horizontal sections, a slight eastward shift may refer to the eastward dip of the fracture planes. The sensitivity of the method allows the detection of crack zones narrower than half a meter, as the muographic method is particularly sensitive to near-vertical density inhomogeneities due to the geometry of the mapping as confirmed by the simulations. In some places, small cavities are also visible along the fracture zones (Fig. 7) due to widened anomalies.

At the edges of the imaged range, especially away from the detectors, a decrease in contrast is noticeable in the parts of the range that are imaged with less overlap (the area outside the focal region).

Note that the density values estimated by tomography, although reflecting the geological variations well, may be biased partly because of the way the discretization is done and partly because of the tomographic technique used. However, the larger fracture zones (probably faults) are clearly visible in a series of horizontal sections (Fig. 7).

**Fig. 8:**
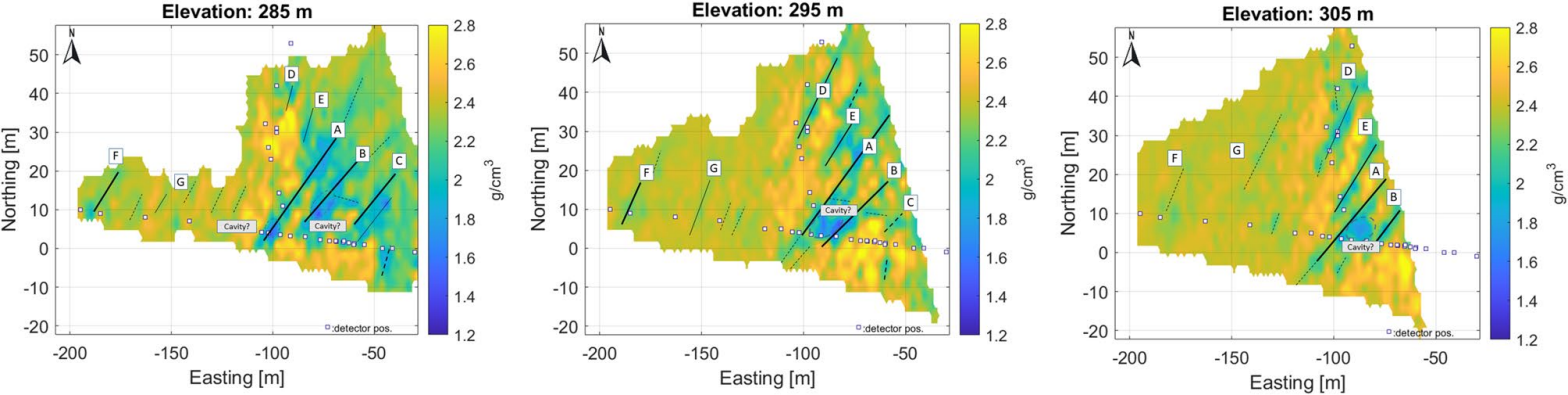
Large scale 3D results on horizontal sections for the whole research area with coarser spatial resolution.

In addition to the high-resolution results for the eastern part shown in the figures above, a lower-resolution overview density map of the entire study area is also presented in Fig. 8 (with a voxel size of 2.5 m).

**Fig. 9:**
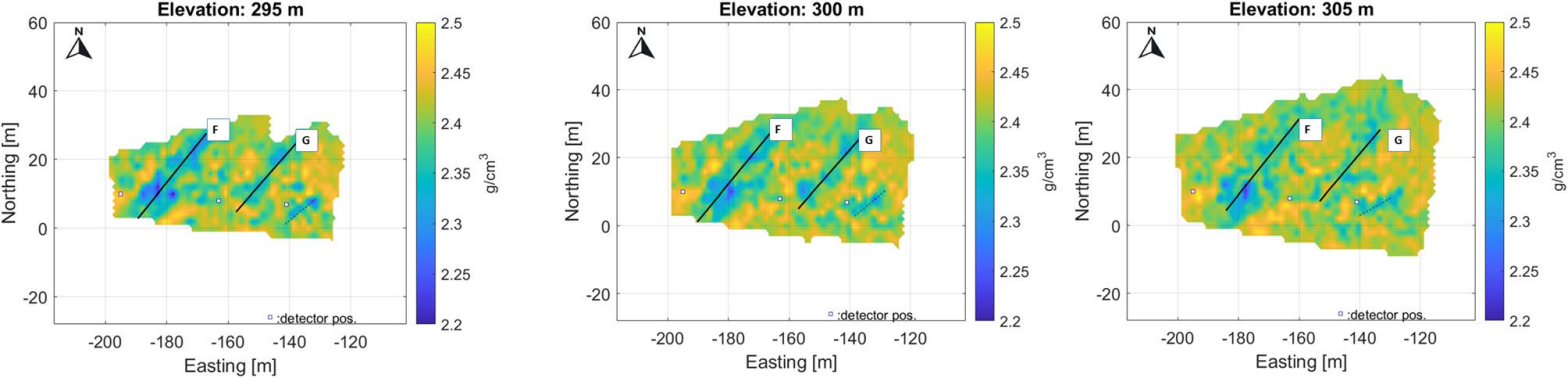
High resolution tomographic from western part of studied area, where the number of measurements is less and the overlap of measurements is poorer and the dolomite thickness is larger, but fracture zones can be identified.

The dominant fracture system is also visible in these tomographic results. Processing on a larger scale results in lower parameter noise levels, but finer details may be lost falling below the detection limit. A larger number of measurements provides less bias and more contrast on the larger-scale overview density map, at least in the eastern part where there is multiple overlap of measurements. Multiscale data processing can be useful for interpretation in displaying information. In the western part, the density dynamics of the reconstruction is smaller due to the larger rock thickness and the poorer coverage of the measurements. In the eastern part, the rock is about 35–70 m thick, while in the western part it is about 70–100 m. Figure 9 shows the tomographic results of the western part with a finer voxel structure.

The finer subdivision makes it possible to track fracture zones more accurately, but due to the low number of measurements, the noise level is significantly higher.

**Fig. 10:**
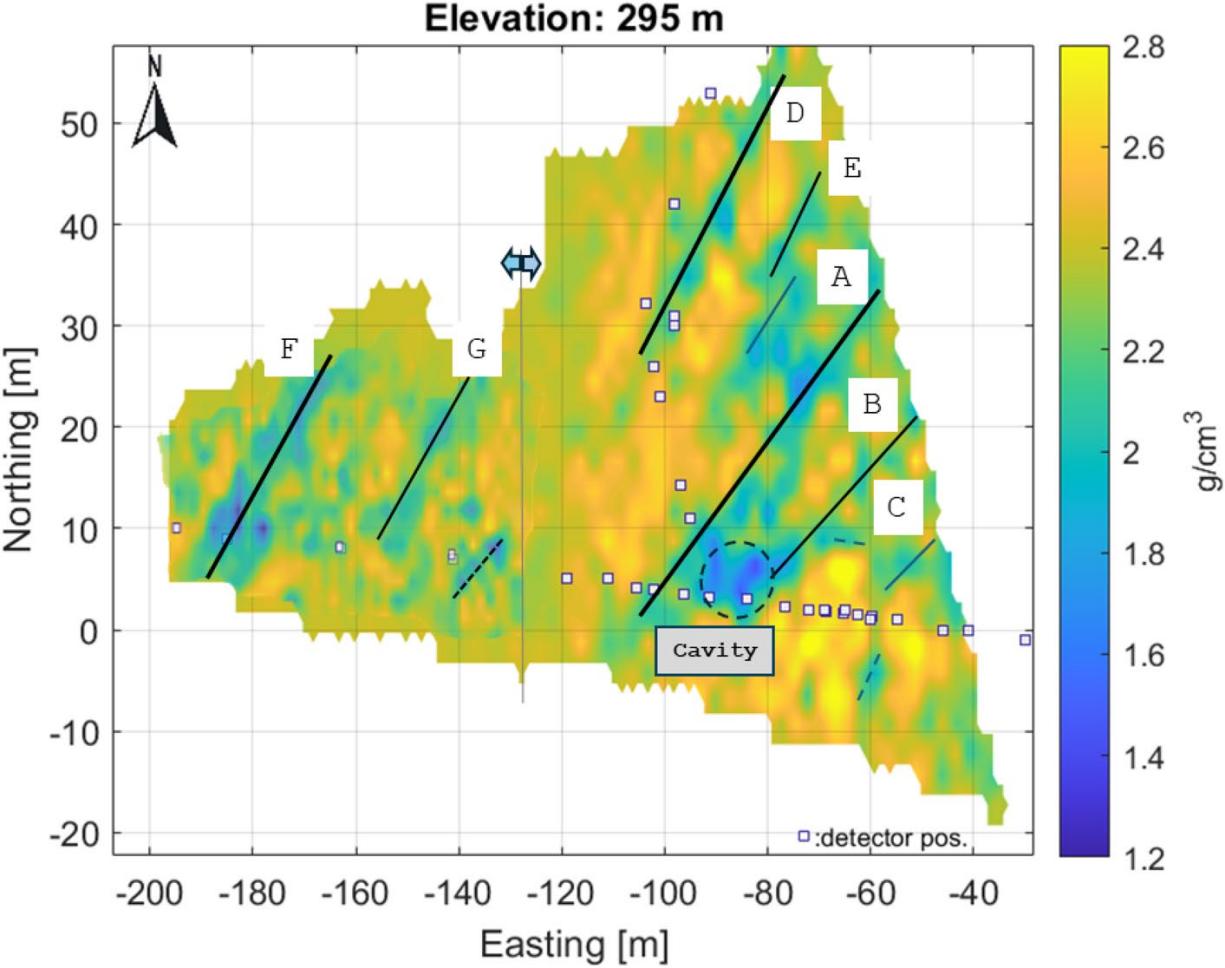
Composite high resolution tomographic results on horizontal section on level 295 m, 25 meters above the detector level. The anomaly of a probable cavity can also be identified here.

The results of the two types of processing were also combined in a composite section (Fig. 10), where the different dynamic range of the density has been equalized.

**Fig. 11:**
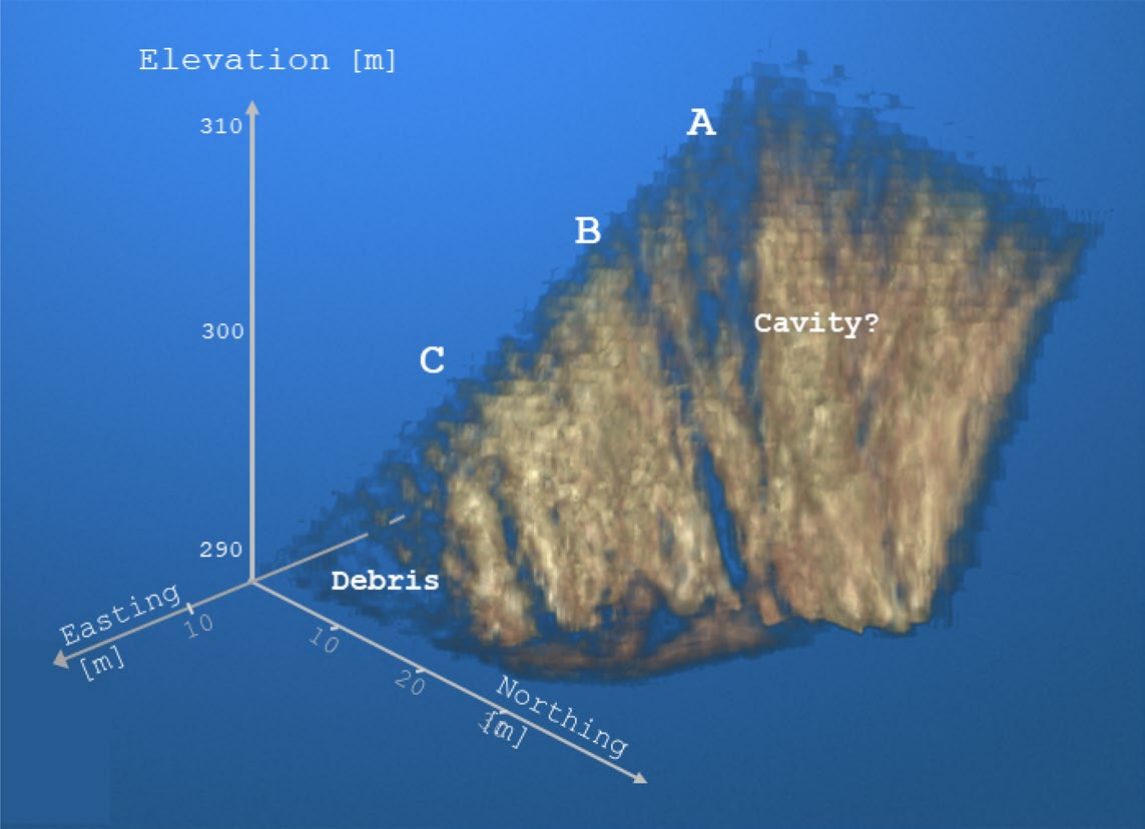
3D view of estimated density reconstruction. View from NE for displaying the main vertical fractures. In the figure, rock regions with higher density (greater than 2.4 g/cm^3^) are highlighted (Image was generated by Matlab volumeViewer version:R2023a .).

For illustrative purposes, the data are also shown in 3D view, looking from NE, because this highlights the dominant fracture zones (Fig. 11).

**Fig. 12:**
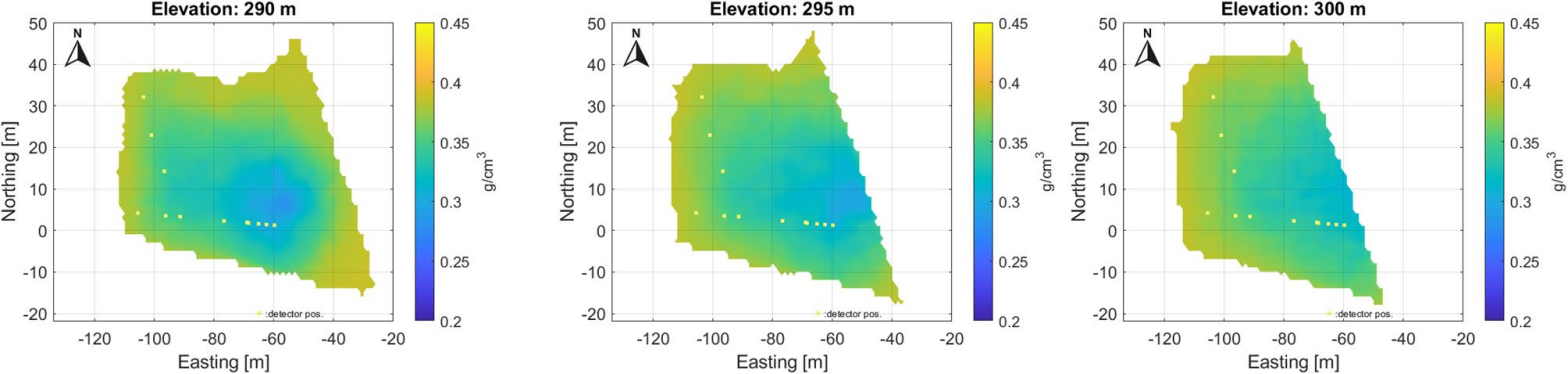
Estimated standard deviation of 3D density distribution reconstruction results on horizontal sections. It can be seen that a standard deviation of 0.25 g/cm^3^ can be achieved in the focal region of the measurement setup, while in the peripheral ranges the standard deviation is close to the prior standard deviation values. Note that the standard deviation alone does not accurately characterize the uncertainty associated with a given voxel element, because the parameter correlation can be high in areas poorly covered by the measurements.

The tomographic reconstruction also includes the determination of the statistical properties of the estimated density distribution. The reliability of the results can be characterized by the standard deviation of the estimated parameters (the square root of the diagonal elements of the estimated covariance matrix). Its spatial distribution is also presented in some horizontal sections (Fig. 12).

### Comparison of tomographic results with data from other sources of information

**Fig. 13:**
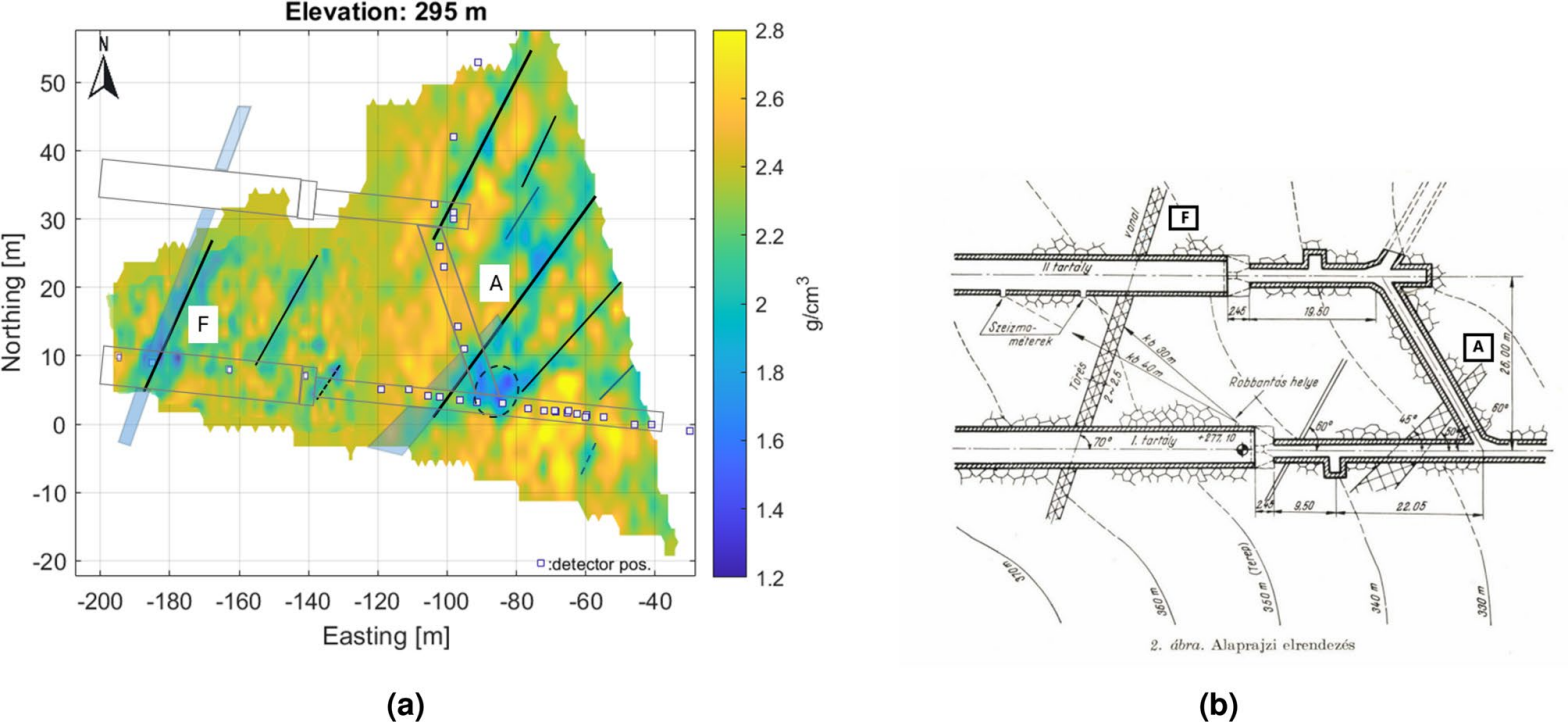
(**a**) Main fracture on the technical drawing and composite horizontal section at level 295 m. The location of the crack zones shows good agreement, (**b**) Original technical drawing^75^.

During the construction of the tunnel, the two main crack zones were detected by the builders and marked on the technical drawings^75^ (Fig. 13). This is an opportunity to partially validate the results. Compared to the composite section, these correspond to low density zones related to fracture zones A and F. The size of the indication corresponds to the thickness ratios indicated in the design drawing. This also confirms the accuracy of muon tomography.

It should be noted that the main passages of the previously discovered hypogene Királylaki cave, which run northeast-southwest under the tunnel, are located under the crack zones marked A and G. The formation of the cave is also related to this fracture system. This cave is connected to the Buda Thermal Karst zone in the Buda Hills.

The tomographic reconstruction also shows the presence of a possible cavity between the main fractures A and B above 295 m (Fig. 7). The shallow drilling of the tunnel ceiling has previously revealed that the identified fractured zones are partially or completely filled with dolomite powder, that is, the fractured zones do not appear at zero density^29^.

### Summary of results

- A unique dense network of muographic measurements was used to map possible fracture networks and cavities in 35 - 100 m thick cherty dolomite layers at Buda Hill.
- The high-resolution measurement data system allowed for a fine tomographic (meter-scale) reconstruction of the rock density distribution.
- The reconstructed image clearly identifies the dominant elements of a near-vertical, predominantly NE-SW crack system.
- The detected apparent fracture system is consistent with the crack zones identified in the excavations and caves.
- The presence of a possible larger cavity was also detected by muon tomography.
- Anomalies that can be interpreted as faults fit well into the geological model of the area.

Due to a dedicated muon telescope with a high angular resolution and 3D tomographic reconstruction on a large data system, the results demonstrate the current potential of subsurface tomography. The method can provide significant aid to geological interpretation even for fracture systems with complex geometries. The results demonstrate that where subsurface detector placement is feasible, it can be a particularly important measurement method, especially in areas where invasive research methods are not applicable (e.g. the area we studied was a nature reserve)

## Discussion

For the tomographic estimates used, the issues of detectability and bias need to be analyzed in detail to judge the validity of the results. Closely related to this is the question of the focal range of the measurement system. The focal range is the part of the geological object with sufficient measurement coverage where the voxel elements are seen by several detectors. In a strict sense, this is the research domain of the muographic system, where reliable measurement information is available and the results are more reliable, with lower variance and little bias. The focal range, bias, and statistical properties of the results can be tested by tomographic estimation on appropriate simulated data (simulated density length vector). For the test, simulated measurements on a simulated geological object were used along with a real detector layout and real topography. The simulations were applied only to the tomographic inversion phase, the largest potential source of error in the whole data processing. The simulations were performed using the real angle dependent variances of the measurement errors in the density length. For the simulation medium models, the rock density was the same as used in the inversion for the bedrock (2.5 g/cm^3^), while for the fracture zones a density value of 1 g/cm^3^ was used, assuming dolomite powder filling (as confirmed by shallow drilling). With the difference between the results and the model, the tomographic process can be tested.

**Fig. 14:**
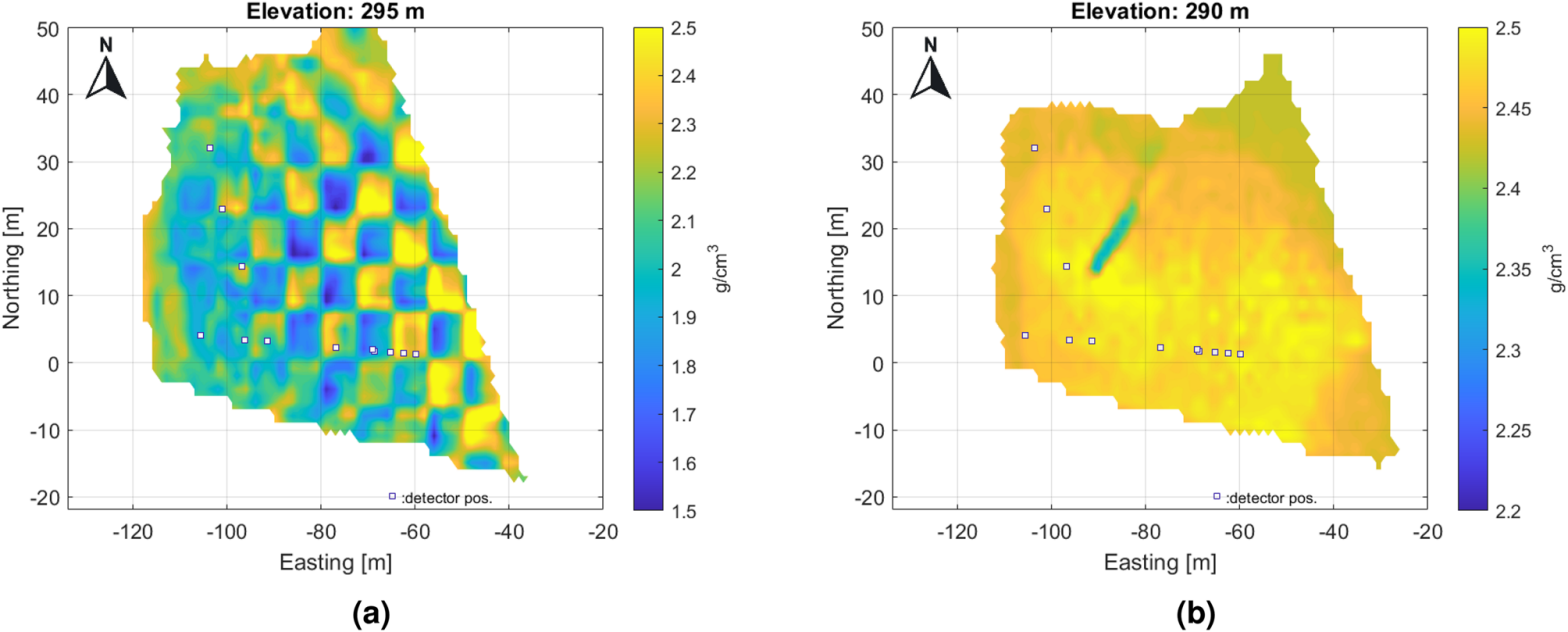
(**a**) The results of the so-called checkerboard test, where the performance of the reconstruction algorithm is investigated assuming a 3D checkerboard-like density distribution. (**b**) This test gives a good indication of the extent of any bias and the focal range of the mapping. Specifically, the sensitivity to the fracture zone is investigated by simulating the reconstruction result for a proper near vertical crack model. Note that the reconstructed distribution shows a smaller artifact shadow towards the NE.

The spatial distribution of the mapping quality is usually examined using a so-called checkerboard test. In this test, the simulated medium is represented as a spatial 3D ”checkerboard” of varying densities. (An example of a horizontal section of the test results is shown in Fig. 14a). The test results clearly identify the fainter zones outside the focal range, where density changes are more significantly biased and the dynamic is suppressed. Note that the geological structure can often be followed outside the focal zone but over a much smaller dynamic range.

The detectability of the fracture zone was investigated using another specific simulation model. In the test, the model simulated a vertical crack with variable parameters (width, height, position, internal density, measurement noise). As an example, a horizontal section of the 3D simulation results is shown in Fig. 14b, where the geological model was a vertical fracture of a half-meter wide in the center of the focal region. The simulation series showed that,even with a width of 0.5 m, a near-vertical crack could be detected in focal position. Note that the detection of horizontal structures is generally subject to a higher bias due to the nature of the mapping (near-vertical scanning). This test can also be used to study the appearance of possible artifacts, which can make the interpretation of results more efficient. Detectability is ultimately determined by the ratio of simulated anomaly to noise. Measurement noise can be kept under control by choosing the right measurement times. In the design of the measurement series, we have tried to keep the relative measurement error to less than a few percent (2-3 percent) for measurements associated with smaller azimuths.

## Methods

In this chapter, the instrumentation and data processing steps involved in tomographic measurements and reconstruction are introduced. (The methodological elements are summarized in a previous study^29^).

### Detectors

**Fig. 15:**
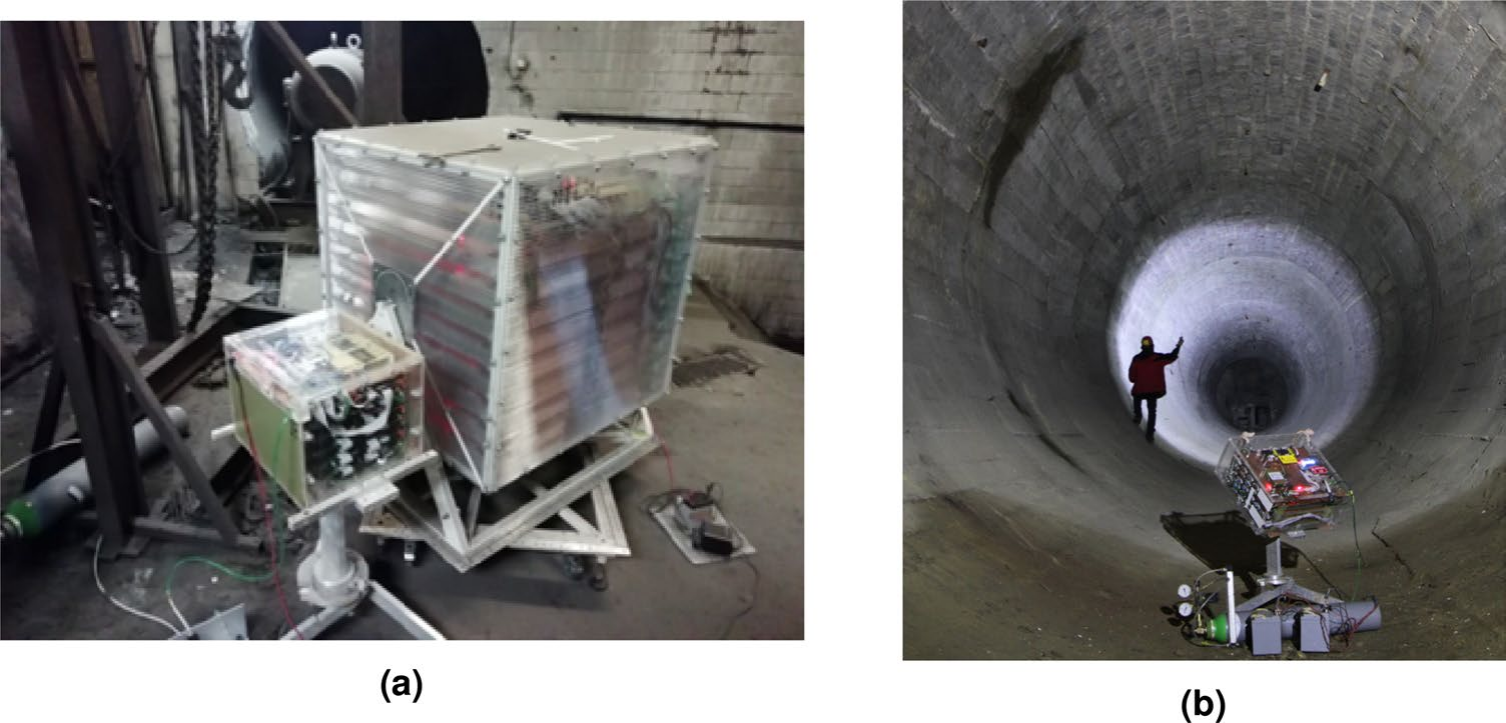
Portable muon detectors of different sensitivities and sizes, designed for field measurements (**a**) Detectors at work. Underground measurements in the Királylaki tunnel (**b**).

The used muograph is a multilayered gaseous tracker designed for field measurements, it is composed of 8 Close Cathode Chambers^74^ with a surface area about 40 × 40 cm^2^ placed equidistantly 40-40 mm apart (illustrated in Fig. 15). Data acquisition and power system are made of costum boards controlled by a RaspberryPi microcomputer, allowing a simple user interface (e.g. via a mobile phone) for managing measurements. The full system is encapsulated in a box of $\approx$ 50 × 50 × 50 cm^3^ and requires 7 W power (usually from standard lead or lithium based batteries).

For a crossing muon each sensitive layer gives hit information in the X and Y directions, and a trigger signal; in case of trigger coincidence from at least 3 chambers the event gets recorded to the local SD-card of the microcomputer. Measurements usually last few weeks, collecting ~ 100,000 individual muon events, allowing good statistics for a broad angle of view. Muon tracks are identified via combinatorial tracking algorithm requiring 6 aligned hits in the 8 chambers, giving tracking efficieny > 98%. Differential efficiencies and performance parameters are extracted form the real data, their time evolution gets monitored to ensure reliability throughout the full survey.

The detector performance is verified in a test environment with a small (0.5m) nearly homogeneous overburden prior to field measurement to ensure uniform angular coverage.

To achieve a good spatial resolution imaging of the geological object, even far away from the detectors, a fine angular resolution (better than 1 degree) is required for the measurements. Imaging requires angular classification (binning) of the data for muon counts and efficiencies. Position resolution of a single CCC layer is $\approx$ 1.5 mm, resulting in better than 5 mrad (0.3 degrees) nominal angular resolution for the muon tracks. Practical angular binning is usually around 1 degree.

The fairly large area of the detectors ensures a high number of detectable muons, allowing a reduction of the measurement time. The detectors can be tilted, thus the measurement can be focused towards the target zone. This is particularly useful for reducing the number of measurements looking at identified regions of interest. The presented equipments are suitable for long measurement campaigns in the field, even in extreme environmental conditions (variable temperature, high humidity, etc.) of mines or caves^76,77^.

Depending on the setting, the muon intensity can be determined in a single measurement position over a range of up to several thousand discrete directions (angular bins) by angularly classifying the recorded muon trajectories. The detectors can be tilted, thus the measurement can be focused towards the target zone. This is particularly useful for reducing the number of measurements after reconnaissance measurements. Our special muograph detectors are suitable for long measurement campaigns in the field, even in extreme environmental conditions (variable temperature, high humidity, etc.) of mines or caves.

#### Muographic survey, detector arrangement

**Fig. 16:**
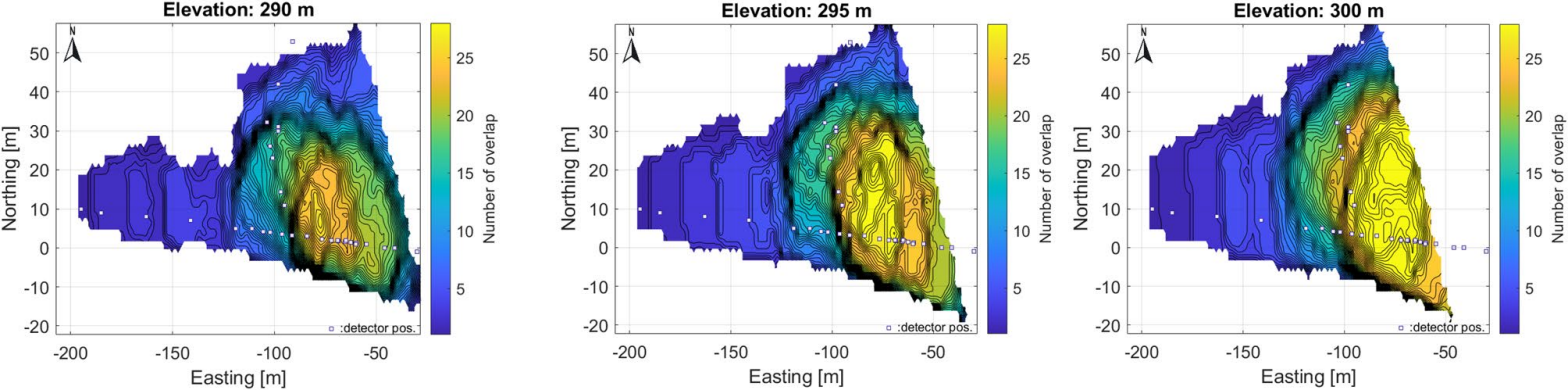
Overlap of mapping cones associated with detectors at different elevation levels. The reliability of the density reconstruction increases for domains with a high overlap number. The best covered areas are seen by more than 30 detectors. This seems to coincide with the results of the chessboard test for the focus area.

Considering the opportunities offered by the environment (tunnel geometry and local topography), an optimal underground measurement setup (system of detector positions and orientation) has been developed, which allows efficient and high resolution tomographic mapping. This also required defining the focal region, the area of the geological object where the best spatial resolution is to be achieved (Fig. 14). During the measurement campaign, the positions of the subsequent measurements were adjusted according to the change in the focus area, based on the previous measurement results. We also aimed to broaden the focus range, if possible. To asses this, a 3D coverage map for the dolomite block under muographic survey was derived, showing how many detector positions are visible in a given volume element of the target zone (Fig. 16).

It can be seen that in a large part of the eastern part, measurements from up to 30 detectors overlap, allowing high-quality, artifact-free tomographic reconstruction. Detectors close to each other improve the resolution of the part of the focal range close to the tunnel, whereas those far away improve mainly the upper part. We have also used the side opening shafts (Fig. 13b) from the main tunnel to improve spatial resolution. The adequacy of the measurement position system has been verified by simulations, mainly from the point of view of the reconstructibility of the density distribution. Optimization is facilitated by the fact that, as mentioned above, the mapping does not depend on changes in rock properties, so that the information gain for a given measurement setup is largely a geometric matter and can be estimated in advance. The sufficiently dense spacing of the detector positions ensured that the density distribution of rocks in the focal range could be reconstructed with a generally good spatial resolution (on the order of meters). The uncertainty (variance) of the measurements depends on the number of muon events recorded in each angular range (according to the Poisson distribution). Hence, the variance of muon intensities can be estimated from counts. This is also reflected in the derived density length error and the reconstructed density distribution error through the propagation of the error. The measurement times at each detector position were set such that the fluctuations (standard deviation) of the time-summated muon events for all orientation angles are kept below a few percentages. The measurement time at a single measurement position was typically 3-4 weeks.

### Reconstruction of rock density distribution

The widely used Bayesian discrete tomography methods have been applied to reconstruct the density distribution of the object studied. Details of the theory^78,79^ of the mathematical background, the algorithm and its application in muography^5,29^ can be found in the literature. Many variants of the method are known^80,81^. In the choice of algorithm, we had to take into account that the nature of the muographic scanning is almost one-sided and the individual muograph measurements cover about a 1.9 steradian cone.

This directional limitation arises as the detector acceptance decreases with the larger angle of incident, while for higher zenith angles, the muon flux decreases while large rock thickness is to be penetrated as well^6^. Important elements of the algorithm are the preliminary assumptions on the geological object under investigation (prior density distribution) and the detailed error model, which includes the measurement errors and also assumptions on the inhomogeneity of the geological object (geological noise).

The tomographic reconstruction is preceded by a complex data processing. In the first phase, the detected muon trajectories are sorted into the appropriate range of direction angles with sufficient resolution for the task. Then, using the direction-dependent efficiency of the detector, a high-resolution muon flux distribution was calculated. The density length for every directional bin can be derived based on the known surface muon flux^15^ and the mechanism of muon energy loss^6^. The relationship between density-length and flux attenuation is described by a monotonic function.

**Fig. 17:**
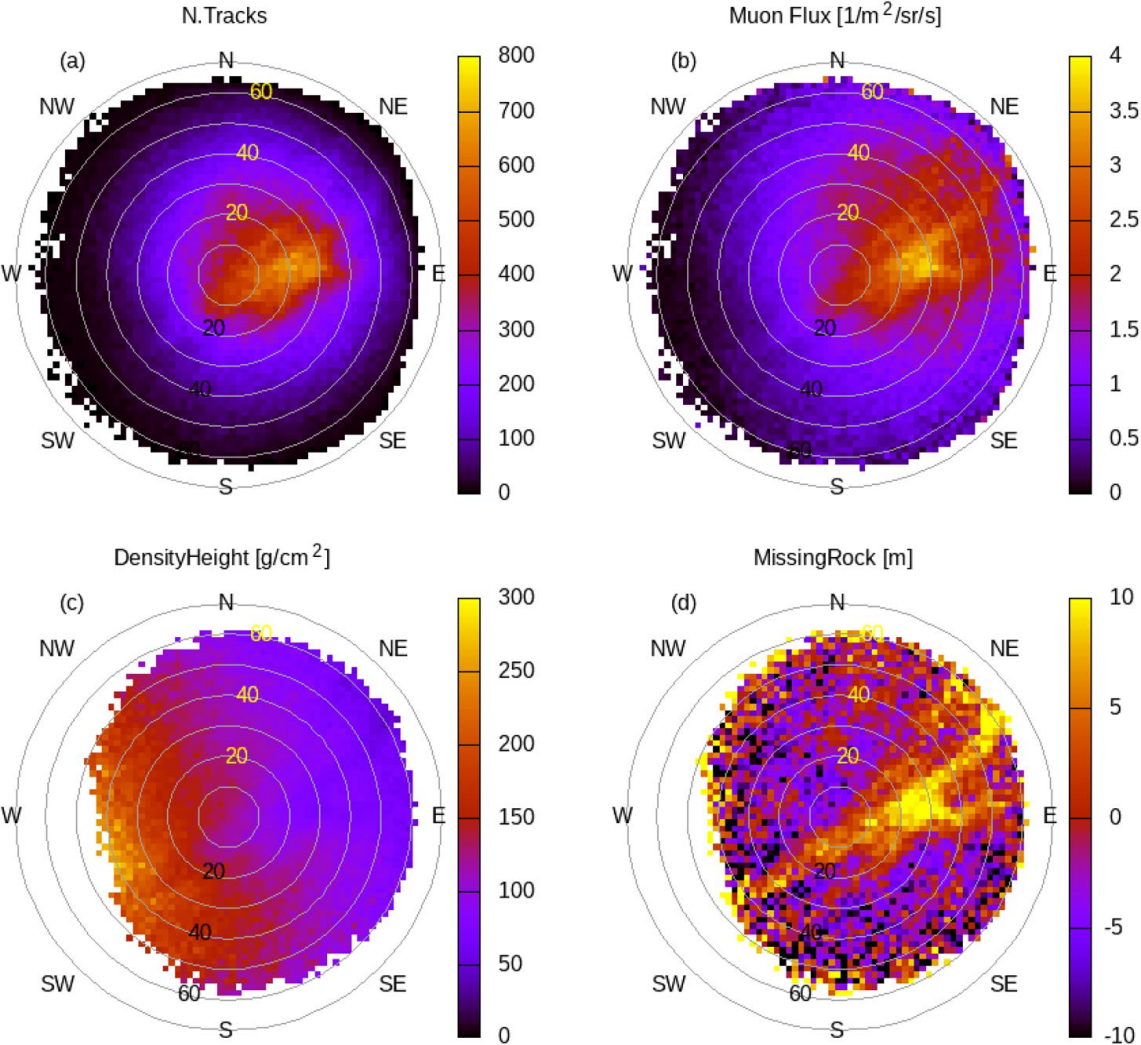
The figure shows the recorded data from the point of view of a detector. Polar plots (binned into 2° cones) showing the number of detected muon tracks (**a**), the computed muon flux (**b**), the converted density-height (**c**), and comparing the latter to the surface data presents the quantity of the missing rock (**d**). This detector was located below fracture A. The significant missing rock anomaly (8–10 m), is associated with the fracture A and the adjacent cavity.

The direction-dependent density-lengths will be the input of the tomographic phase for the density reconstruction. Another important input to tomography is the error distribution parameters associated with the density length. This distribution is obtained by propagating the distribution of measurement errors. This error is particularly large in the case of high azimuth-angle bins. The partial results of the data processing and its directional distributions are shown in a series of polar plots from the detector’s point of view (Fig. 17). The selected example illustrates how a cavity-related anomaly is reflected in the partial results (muon counts, muon flux, density-length and calculated missing rock length).

In the case of cavities and fracture zones, there is a high density contrast between the rock and the partially or completely filled cracks and cavities. This is the main source of the density anomalies in the study area. For the reconstruction, the investigated domain (the “scanned”) (rock body) was divided into disjoint voxels (the uniform 3D volume element defined in the geographic frame), whose density is now considered homogeneous. The results as the estimated density distribution are represented on this voxel-base. The voxel structure and the dimensions of the voxel also determine the resolution at which the density distribution of the geological object is approximated.

A voxel structure that is too fine-grained can also increase the uncertainty of the results because of the strong correlation between estimated voxel densities, which is an expression of equivalent cases. The goodness of tomographic reconstruction is influenced by the measurement setup, topography, and voxel structure. Based on these, we can optimize the measurement system and the parameters of the tomographic reconstruction.

#### Information gain

The measurement sensitivity of a given voxel can be determined, in a first approximation, from the section of possible muon trajectories in a voxel arriving at the detector position at a given angle. This characterizes the information that can be obtained for a given volume of rock^29^. From the tomography point of view, this information is summed up for all survey measurements, weighted by the reciprocal of the estimated variance of the density length derived from the measurement (Fisher-information^82^). Due to the linearity of the tomographic problem, a sensitivity map can be prepared in advance for the current detector layout without knowing the properties of the rock. This will help to judge the goodness of the measurement design and interpret the results.

#### Estimation of density distribution

Due to the “projective” nature of muography, the reconstruction of the density distribution is not unambiguous (at least in part of the study area, where measurement coverage is poor), which makes the basic estimation problem mathematically unsolvable (singular, ill posed problem)^78,79^, which can be solved by regularization^83^, whereby our prior knowledge about the geological structure can be incorporated into the estimation operation, as a prior density model (Bayesian estimation)^29^.

The prior is assumed to be Gaussian distributed (with density vector $\varvec{\rho _0}$ and covariance matrix $C_0$) The starting point of the linearized Bayesian estimation (inversion) is the density length vector (**y**) obtained by the transformation of the detected angular muon counts during the preprocessing. The density length vector can be written as a linear transformation the true (unknown) voxelized density distribution vector ($\rho$):

1 $$\begin{aligned} {{\textbf {y}}}={{\textbf {F}}}\varvec{\rho } \end{aligned}$$

The Jacobian (**F**) is derived from the voxelization of the muon trajectory field associated with the detector (Fig. 2a) The regularized estimation for the voxelized density distribution:

2 $$\begin{aligned} \hat{\varvec{\rho }}=({{\textbf {R}}}+{{\textbf {C}}}_0^{-1})^{-1}({{\textbf {F}}}^{T}{{\textbf {C}}}_{y}^{-1} {{\textbf {y}}}+{{\textbf {C}}}_0^{-1}\varvec{\rho }_0) \end{aligned}$$

Where R is the Fisher information matrix of the problem (${{\textbf {R}}}={{\textbf {F}}}^T {{\textbf {C}}}_y^{-1}{{\textbf {F}}}$).

The Bayesian approach is formally equivalent to regularization. Heuristically, we can say that the procedure combines the information from the measurements and the prior assumption, weighted according to the reliability of the information to produce the final estimate. The cost of this is that the estimated density values may become biased, although their spatial distribution over the focal range still reflects density variations, so that the result is still useful in geological interpretation even without correction. The regularization that allows tomographic reconstruction in this case is the preliminary assumption that the volume of the screened rock body predominantly has a well-defined density (porous dolomite density: 2.5 g/cm^3^) and that the proportion of fractured lower density domains is relatively small. Based on the latter assumption and on the knowledge of the inhomogeneity of the rock, the covariance matrix of the prior distribution is determined. In the calculations, the standard deviation of the prior density values was 0.4 g/cm^3^. (weakly informative prior). It is important to note that the prior distribution does not refer to the rock in general but to the voxelized rock. (The traditional regularization parameter can correspond to the reciprocal of the prior distribution variances, but in the form given here it can be tuned spatially.) The estimation formula compares the prior standard deviation with the measurement information (Fisher information), so the choice of the prior is used to set the limit of acceptance of the measurement information. This implies a dependence of the prior variance on the voxel size. Note that the wrong choice of the prior density distribution creates a characteristic ’frame’ around the focal region. Regularization has a stronger influence on the results for the spatial domains for which we have little and uncertain measurement information. This is generally the case at the edges of the volume under investigation. Simulation also plays an important role in determining the parameterization of the tomographic calculations In the focal area of the survey, the measurement information suppresses the distortion effect of the prior.

For structures smaller than the voxel size, the bias is even more significant because the fitting is done to determine the characteristic density for the entire voxel, so the detectability of small structures decreases with size and disappears when the anomaly reaches the noise level.

#### Uncertainty and limitation

The results of muographic data processing and tomographic reconstruction are subject to a number of sources of error, which must be considered when estimating the statistical properties of the results. The result vector inherits the errors of the previous complete data acquisition and processing. The bias vector (b) on the expected value of the result vector (based on Eq. 2) is the following^29^:

3 $$\begin{aligned} {{\textbf {b}}}=({{\textbf {R}}}+{{\textbf {C}}}_0^{-1})^{-1} ({{\textbf {C}}}_0^{-1}(\varvec{\rho }_0 -\varvec{\rho })+{{\textbf {F}}}^T {{\textbf {C}}}_y^{-1}{\delta }{{\textbf {y}}}) \end{aligned}$$

where ${\delta }y$ is the systematic error vector in density length vector, and ${\rho }$ is the (unknown) true density. Reconstruction can be made more efficient by choosing a more accurate density model, since the bias depends on the difference between the real and the a priori density vector (Eq. 3).

The estimated covariance matrix (${{\textbf {C}}}_{\rho }$) characterizing the distribution of the zero-mean error component of the estimated density vector (also based on Eq. 2):

4 $$\begin{aligned} {{\textbf {C}}}_{\rho }=({{\textbf {R}}}+{{\textbf {C}}}_0^{-1})^{-1} \end{aligned}$$

This error term inherits the errors of the previous processing steps through the Fisher information matrix, which contains the covariance matrix of the binned density lengths. The error model used in the tomographic procedure is rather complex. The dominant component is the statistical error of the binned measurements, which is also reflected in the density-length error (${{\textbf {C}}}_y$) through error propagation. The other statistical error components (muon flux temporal fluctuations, background process etc.) are negligible. The systematic error components (efficiency bias, topography (DEM) error, muon flux transformation function error) that also load on the density length are kept low (negligible). The error in the direction angle of the muon trajectories is dominated by a multiple scattering effect^8,10^ (the fitting error in the muon track parameters is significantly smaller). The minimum aperture of the bin angle is determined by this error component. The error term due to geometry simplification (point detector approximation, trajectory length fluctuations within the mapping cone, etc.) is negligible. The effect of the listed components may be reflected in the error of the tomographic results according to Eq. 3 and 4.

The above-mentioned sources of error, the detector specifications and the measurement environment (target object size), can be used to judge the limitations of the method. In the case of density reconstruction, the most important question is spatial resolution within the volume of interest. For a detector, the sensitivity cones associated with the angular bins indicate the appropriate voxel dimensions to be used at various distances from the device. For apertures smaller than 10-15 mrad, the neighboring bins will be correlated. This appears in the covariance matrix of the measured data, further increasing the instability of the estimation method.

The measurements presented in this paper demonstrated that both statistical and systematic errors of three-dimensional density reconstruction can be controlled with a sufficient number of measurement points, sufficient statistics and resolution.

## Data Availability

Correspondence and reasonable requests for materials and data should be addressed to L.B.
